# Supplementary material for: SEL1L–HRD1 endoplasmic reticulum-associated degradation controls STING-mediated innate immunity by limiting the size of the activable STING pool
Source: Nat Cell Biol. 2023 May 4;25(5):726–39. doi: 10.1038/s41556-023-01138-4 (PMC10185471; doi:10.1038/s41556-023-01138-4)
Supplement: Supplementary file 1 — Reporting Summary [file 41556_2023_1138_MOESM1_ESM.pdf]

nature portfolio

Corresponding author(s): Ling Qi; Yewei Ji

Last updated by author(s): Feb 16, 2023

Reporting Summary

Nature Portfolio wishes to improve the reproducibility of the work that we publish. This form provides structure for consistency and transparency in reporting. For further information on Nature Portfolio policies, see our Editorial Policies and the Editorial Policy Checklist.

Statistics

For all statistical analyses, confirm that the following items are present in the figure legend, table legend, main text, or Methods section.

n/a	Confirmed	
☐	☒	The exact sample size (*n*) for each experimental group/condition, given as a discrete number and unit of measurement
☐	☒	A statement on whether measurements were taken from distinct samples or whether the same sample was measured repeatedly
☐	☒	The statistical test(s) used AND whether they are one- or two-sided<br>*Only common tests should be described solely by name; describe more complex techniques in the Methods section.*
☐	☒	A description of all covariates tested
☐	☒	A description of any assumptions or corrections, such as tests of normality and adjustment for multiple comparisons
☐	☒	A full description of the statistical parameters including central tendency (e.g. means) or other basic estimates (e.g. regression coefficient) AND variation (e.g. standard deviation) or associated estimates of uncertainty (e.g. confidence intervals)
☐	☒	For null hypothesis testing, the test statistic (e.g. *F*, *t*, *r*) with confidence intervals, effect sizes, degrees of freedom and *P* value noted<br>*Give P values as exact values whenever suitable.*
☒	☐	For Bayesian analysis, information on the choice of priors and Markov chain Monte Carlo settings
☒	☐	For hierarchical and complex designs, identification of the appropriate level for tests and full reporting of outcomes
☒	☐	Estimates of effect sizes (e.g. Cohen's *d*, Pearson's *r*), indicating how they were calculated

Our web collection on statistics for biologists contains articles on many of the points above.

Software and code

Policy information about availability of computer code

Data collection	Confocal data of STING localization were collected using NIS-elements C 4.60.00 (Nikon) and stedycon smart control (STEDYCON super-resolution microscopy); H&E images of liver and adipose tissue were collected using Aperio Imagescope software v102.0.4.6; Flow data were collected using the FACSDiva v6.2 software (BD Biosciences); Western Blot data were collected by Image Lab software 4.1 (Bio-rad); TEM images were collected with the use of JEM-1400 TEM.
Data analysis	Prism v6 and v8 was used for statistics analysis; Imaging data of STING colocalization were analyzed using the Fiji 2.0.0 software (ImageJ); Flow data were analyzed using the FACSDiva v6.2 software (BD Biosciences) and Flowjo 8.7 (Flowjo.com); Western Blot data were analyzed by Image Lab software 4.1 (Bio-rad). TEM images were analyzed with Image-Pro Plus 6.0 software.

For manuscripts utilizing custom algorithms or software that are central to the research but not yet described in published literature, software must be made available to editors and reviewers. We strongly encourage code deposition in a community repository (e.g. GitHub). See the Nature Portfolio guidelines for submitting code & software for further information.

Data

Policy information about availability of data

All manuscripts must include a data availability statement. This statement should provide the following information, where applicable:

- Accession codes, unique identifiers, or web links for publicly available datasets
- A description of any restrictions on data availability
- For clinical datasets or third party data, please ensure that the statement adheres to our policy

Previously published STING proximity-based proteomics data are available from [Chu, TT., Tu, X., Yang, K. et al. Nature (2021). https://doi.org/10.1038/s41586-021-03762-2]. All source data for all graphical and unprocessed blot images are provided with this study. All other data supporting the findings of this study are available from the corresponding author on reasonable request.

Human research participants

Policy information about studies involving human research participants and Sex and Gender in Research.

Reporting on sex and gender	N/A
Population characteristics	N/A
Recruitment	N/A
Ethics oversight	N/A

Note that full information on the approval of the study protocol must also be provided in the manuscript.

Field-specific reporting

Please select the one below that is the best fit for your research. If you are not sure, read the appropriate sections before making your selection.

☒ Life sciences ☐ Behavioural & social sciences ☐ Ecological, evolutionary & environmental sciences

For a reference copy of the document with all sections, see nature.com/documents/nr-reporting-summary-flat.pdf

Life sciences study design

All studies must disclose on these points even when the disclosure is negative.

Sample size	Based on sample size formula of the power analysis, N=8(CV)2[1+(1-PC)2]/(PC)2, to reach the error = 0.05, Power = 0.80, percentage change in means (PC) = 20%, co-efficient of variation (CV) = 10 ~ 15% (varies between the experiments), 4-6 mice per group are the minimal number of mice to obtain statistical significance and to ensure adequate power.<br>The sample size for each animal experiment was described in the figure legend. The way of predetermining sample size is described in Supplementary materials page 1, "Power analysis of the animal size".<br>No sample size calculation was performed for in vitro experiments, sample size of these experiments was determined based on previously published studies in the field of cell biology and immunology where differences were observed. Sample sizes were provided throughout manuscript.
Data exclusions	No animals or samples were excluded from the analysis.
Replication	All experiments were repeated at least twice or performed with independent samples. All attempts for replication were successful.<br>This is described in text methods part, section "Statistical Analysis". The exact repeat times of experiments are indicated in the figure legends.
Randomization	For LPS injection, DMXAA injection, HFD feeding experiments, mice were sex- and age-matched and randomly assigned to experimental groups according to the genotype. For secretome treatment experiments, mice with similar tumor volume and body weights following tumor cell implantation were randomized into different groups of treatment. For ligands and chemical molecules treatment experiments, cell culture samples were randomly allocated to control and experimental groups.
Blinding	When experiments were done by one investigator, blindness is not applied or possible. When experiments are done sequentially by different investigators, investigators were blinded to allocation during experiments and outcome assessment. The experiments were repeated by different investigators independently.

Reporting for specific materials, systems and methods

We require information from authors about some types of materials, experimental systems and methods used in many studies. Here, indicate whether each material, system or method listed is relevant to your study. If you are not sure if a list item applies to your research, read the appropriate section before selecting a response.

Materials & experimental systems

n/a	Involved in the study
☐	☒ Antibodies
☐	☒ Eukaryotic cell lines
☒	☐ Palaeontology and archaeology
☐	☒ Animals and other organisms
☒	☐ Clinical data
☒	☐ Dual use research of concern

Methods

n/a	Involved in the study
☒	☐ ChIP-seq
☐	☒ Flow cytometry
☒	☐ MRI-based neuroimaging

Antibodies

Antibodies used

Flow cytometric antibodies for cell markers: CD4 (GK1.5, BioLegend 100408), CD8 (YTS169.4, Thermo Fisher MA5-17605, MA5-17607), F4/80 (BM8, BioLegend 123116, 123114), CD11b (M1/70, BioLegend 101206), Gr-1 (RB6-8C5, BioLegend 108408), TCR β (H57-597, BioLegend 109206), B220 (RA3-6B2, BioLegend 103206, 103208), CD45 (30-F11, BioLegend 103130), I-A/I-E (M5/114.15.2, BioLegend 107645, 107608), H-2Kb/H-2Db (28-8-6, AF6-88.5, BioLegend 114606, 116506), TLR2 (CB225, BioLegend 148604), TLR4 (SA15-21, BioLegend 145406), PD-L1 (10F.9G2, BioLegend 124308) and anti-CD16/CD32 (93, BioLegend 101302). All used at 1:100 or 200 dilution.
Western blot antibodies: HSP90 (Abcam ab13492, 1:6,000), β-Tubulin (Proteintech 10068-1-AP, 1:3000), Caspase-3 (Cell Signaling 8G10, 1:1,000), β-Actin (Proteintech 20536-1-AP, 1:3000), IκBα (Cell Signaling 9242, 1:2,000), SEL1L (Abcam ab78298, 1:1000), BiP (Abcam ab21685; 1:5000), HRD1 (Dr. Richard Wojcikiewicz, 1:300; Proteintech 13473-1-AP, 1:1000), STING (Proteintech 19851-1AP, 1:1500; Cell Signaling D2P2F; 1:2000), p-STING (Ser365) (Cell Signaling D8F4W; 1:2000), cGAS (Cell Signaling D3080; 1:2000), p-TBK1 (Ser172) (Cell Signaling D52C2; 1:1000), TBK1 (Cell Signaling E9H5S; 1:2000), p-IRF-3 (Ser396) (Cell Signaling D601M; 1:2000), IRF-3 (Cell Signaling D83B9; 1:2000), ATG7 (Cell Signaling D12B11; 1:1000), OS9 (Abcam ab109510; 1:3000), eIF2α (Cell Signaling 9722; 1:2000), p-eIF2α (Cell Signaling 3597S; 1:2000), IRE1α (Cell Signaling 3294; 1:3000), ERP44 (Cell Signaling 2886; 1:3000), STIM1 (Cell Signaling 4916; 1:2000), HA (SIGMA H3663; 1:2000), c-Myc (SIGMA C3956; 1:2000), Flag (SIGMA F1804; 1:2000), H2A (Cell Signaling 2578; 1:5000), LC3B (Cell Signaling 2775; 1:2000), PDI (ENZO ADI-SPA-890, 1:2,000), Ubiquitin (Santa Cruz P4D1, 1:200), SOAT1 (GeneTex GTX32890, 1:1,000), FACL4 (Abcam ab155282, 1:1,000), Calnexin (Proteintech 10427-2-AP, 1:20,000), goat anti-rabbit IgG-HRP (BioRad 1721019, 1:5,000) and goat anti-mouse IgG-HRP (BioRad 1721011, 1:5,000).
Antibodies for immunofluorescent staining: STING (Proteintech 19851-1AP, 1:200), KDEL (Abcam MAC 256, 1:200), Phospho-STING (Ser365) (Cell Signaling D1C4T, 1:200), TGN38 (Santa Cruz sc-166594,1:200), CD63 (Santa Cruz sc-5275,1:200), LAMP1 (DSHB 1D4B, 1:50).
Antibodies for immunoprecipitation: STING (Proteintech 19851-1AP), SEL1L (Abcam ab78298), protein A-agarose beads (Invitrogen 20334), agarose-conjugated anti-FLAG (Sigma A4596), agarose-conjugated anti-Myc (Sigma 16-219) , streptavidin agarose (Thermo Fisher 20353). 1μg antibody or 30 μl agarose beads for 1mL sample lysis.

Validation

Antibodies used in this study were from best available vendors with good citation and validated by the vendors. We further verified specificity busing Western blot, IP and confocal imaging, compared with isotype controls as well as positive and negative control samples. The information of validation and citation are available on the manufacturer's websites.
1) Mouse anti-HSP90 (ab13492/ Abcam, 1:6,000): The manufacture states that the specificity of the antibody was tested by western blot on various mammalian cell lysates. https://www.abcam.com/hsp90-antibody-ac88-ab13492.html#lb
2) Rabbit β-Tubulin (Proteintech 10068-1-AP, 1:3000): The manufacture states that the specificity of the antibody was tested by western blot on various mammalian cell lysates. https://www.ptglab.com/products/TUBB3-Antibody-10068-1-AP.htm
3) Rabbit anti- Caspase-3 (Cell Signaling 9665, 1:1,000): The manufacture states that the specificity of the antibody was tested by western blot on HeLa cell lysates. https://www.cellsignal.com/product/productDetail.jsp?productId=9665
4) Rabbit anti- β-Actin (Proteintech 20536-1-AP, 1:3000): The manufacture states that the specificity of the antibody was tested by western blot on various mammalian cell lysates and tissues. https://www.ptglab.com/products/ACTB-Antibody-20536-1-AP.htm
5) Rabbit anti-IκBα (Cell Signaling 9242, 1:2,000): The manufacture states that the specificity of the antibody was tested by western blot on HeLa cell lysates. https://www.cellsignal.com/products/primary-antibodies/ikba-antibody/9242?_=1673953782135&Ntt=9242&tahead=true
6) Rabbit anti- SEL1L (Abcam ab78298, 1:1000; Abclonal A12073, 1:2000): The manufacture states that the specificity of the antibody was tested by western blot on various mammalian cell lysates and tissues. https://www.abcam.com/sel1l-antibody-ab78298.html#lb, https://abclonal.com/search/index?keyword=SEL1L+Rabbit+pAb&catid=56&__hash__=d54593da28f3677abb7adc59204265a1_e4843318fa4fda248241fe6564807c09&Searchbar=
7) Rabbit anti- BiP (Abcam ab21685; 1:5000): The manufacture states that the specificity of the antibody was tested by western blot on various mammalian cell lysates. https://www.abcam.com/grp78-bip-antibody-ab21685.html#lb
8) Rabbit anti-HRD1 (Proteintech 13473-1-AP, 1:1000): The manufacture states that the specificity of the antibody was tested by western blot on various mammalian cell lysates and tissues. https://www.ptglab.com/products/SYVN1-Antibody-13473-1-AP.htm
9) Rabbit anti- STING (Proteintech 19851-1AP, 1:1500 for WB): The manufacture states that the specificity of the antibody was tested by western blot on various mammalian cell lysates and tissues. https://www.ptglab.com/products/TMEM173-Antibody-19851-1-AP.htm
10) Rabbit anti-p-STING (Ser365) (Cell Signaling 72971 clone D8F4W, 1:2000): The manufacture states that the specificity of the antibody was tested by western blot on Raw 264.7 cells. https://www.cellsignal.com/products/primary-antibodies/phospho-sting-

ser365-d8f4w-rabbit-mab/72971?site-search-type=Products&N=4294956287&Ntt=d8f4w&fromPage=plp&_requestid=10805356
11) Rabbit anti-cGAS (Cell Signaling 31659 clone D3080; 1:2000): The manufacture states that the specificity of the antibody was tested by western blot on various mammalian cell lysates. https://www.cellsignal.com/products/primary-antibodies/cgas-d3o8o-rabbit-mab-mouse-specific/31659?site-search-type=Products&N=4294956287&Ntt=cgas&fromPage=plp
12) Rabbit anti- p-TBK1 (Ser172) (Cell Signaling 5483 clone D52C2; 1:1000): The manufacture states that the specificity of the antibody was tested by western blot on THP-1 cell lysates. https://www.cellsignal.com/products/primary-antibodies/phospho-tbk1-nak-ser172-d52c2-xp-rabbit-mab/5483?site-search-type=Products&N=4294956287&Ntt=d52c2&fromPage=plp
13) Validation of Rabbit anti- TBK1 (Cell Signaling 51872 clone E9H5S; 1:2000): The manufacture states that the specificity of the antibody was tested by western blot on various mammalian cell lysates. https://www.cellsignal.com/products/primary-antibodies/tbk1-nak-e9h5s-mouse-mab/51872?site-search-type=Products&N=4294956287&Ntt=e9h5s&fromPage=plp&_requestid=10806496
14) Validation of Rabbit anti- p-IRF-3 (Ser396) (Cell Signaling 29047 clone D601M; 1:2000): The manufacture states that the specificity of the antibody was tested by western blot on Human cell lysates and mice. https://www.cellsignal.com/products/primary-antibodies/phospho-irf-3-ser396-d6o1m-rabbit-mab/29047
15) Validation of Rabbit anti- IRF-3 (Cell Signaling 4302 clone D83B9; 1:2000): The manufacture states that the specificity of the antibody was tested by western blot on various mammalian cell lysates. https://www.cellsignal.com/products/primary-antibodies/irf-3-d83b9-rabbit-mab/4302?site-search-type=Products&N=4294956287&Ntt=anti-+irf-3+&fromPage=plp
16) Validation of Rabbit anti- ATG7 (Cell Signaling 8558 clone D12B11; 1:1000): The manufacture states that the specificity of the antibody was tested by western blot on various mammalian cell lysates. https://www.cellsignal.com/products/primary-antibodies/atg7-d12b11-rabbit-mab/8558?site-search-type=Products&N=4294956287&Ntt=atg7&fromPage=plp
17) Validation of Rabbit anti- OS9 (Abcam ab109510; 1:3000): The manufacture states that the specificity of the antibody was tested by western blot on various mammalian cell lysates. https://www.abcam.com/os9-antibody-epr42722-ab109510.html
18) Validation of Rabbit anti- eIF2α (Cell Signaling 9722; 1:2000): The manufacture states that the specificity of the antibody was tested by western blot on PC12 cell lysates. https://www.cellsignal.com/products/primary-antibodies/eif2a-antibody/9722
19) Validation of Rabbit anti- p-eIF2α (Cell Signaling 3597; 1:2000): The manufacture states that the specificity of the antibody was tested by western blot on various mammalian cell lysates. https://www.cellsignal.com/products/primary-antibodies/phospho-eif2a-ser51-119a11-rabbit-mab/3597?_=1673956082958&Ntt=3597S&tahead=true
20) Validation of Rabbit anti- IRE1α (Cell Signaling 3294 clone 14C10; 1:3000): The manufacture states that the specificity of the antibody was tested by western blot on various mammalian cell lysates. https://www.cellsignal.com/products/primary-antibodies/ire1a-14c10-rabbit-mab/3294
21) Validation of Rabbit anti- ERP44 (Cell Signaling 2886; 1:3000): The manufacture states that the specificity of the antibody was tested by western blot on various mammalian cell lysates. https://www.cellsignal.com/products/primary-antibodies/erp44-antibody/2886
22) Validation of Rabbit anti- STIM1 (Cell Signaling 4916; 1:2000): The manufacture states that the specificity of the antibody was tested by western blot on various mammalian cell lysates. https://www.cellsignal.com/products/primary-antibodies/stim1-antibody/4916
23) Validation of Mouse anti- HA (Sigma H3663; 1:2000): The manufacture states that the specificity of the antibody was tested by western blot on HEK-293T cell lysates. https://www.sigmaaldrich.com/US/en/product/sigma/h3663
24) Validation of Rabbit anti- c-Myc (Sigma C3956; 1:2000): The manufacture states that the specificity of the antibody was tested by western blot on HEK-293T cell lysates. https://www.sigmaaldrich.com/US/en/product/sigma/c3956
25) Validation of Mouse anti- Flag (Sigma F1804 clone M2; 1:2000): The manufacture states that the antibody has been optimized for signal band detection of the FLAG antigen (DYKDDDK) fused proteins in mammalian, plant and bacterial systems. https://www.sigmaaldrich.com/US/en/product/sigma/f1804
26) Validation of Rabbit anti- H2A (Cell Signaling 2578; 1:5000): The manufacture states that the specificity of the antibody was tested by western blot on various mammalian cell lysates. https://www.cellsignal.com/products/primary-antibodies/histone-h2a-antibody-ii/2578
27) Validation of Rabbit anti- LC3B (Cell Signaling 2775; 1:2000): The manufacture states that the specificity of the antibody was tested by western blot on various mammalian cell lysates. https://www.cellsignal.com/products/primary-antibodies/lc3b-antibody/2775
28) Validation of Rabbit anti- PDI (ENZO ADI-SPA-890, 1:2,000): The manufacture states that the specificity of the antibody was tested by western blot on various mammalian cell lysates and tissues. https://www.enzolifesciences.com/ADI-SPA-890/pdi-polyclonal-antibody/
29) Validation of Mouse anti- Ubiquitin (Santa Cruz sc8017 clone P4D1, 1:200): The manufacture states that the specificity of the antibody was tested by western blot on various human cell lysates. https://www.scbt.com/p/ubiquitin-antibody-p4d1?requestFrom=search
30) Validation of Rabbit anti- SOAT1 (GeneTex GTX32890, 1:1,000): The manufacture states that the specificity of the antibody was tested by western blot on various mammalian cell lysates and tissues. https://www.genetex.com/Product/Detail/SOAT1-antibody/GTX32890#datasheet
31) Validation of Rabbit anti- FACL4 (Abcam ab155282, 1:1,000): The manufacture states that the specificity of the antibody was tested by western blot on various mammalian cell lysates and tissues. https://www.abcam.com/facl4-antibody-epr8640-ab155282.html#lb
32) Validation of Rabbit anti- Calnexin (Proteintech 10427-2-AP, 1:20,000): The manufacture states that the specificity of the antibody was tested by western blot on various mammalian cell lysates. https://www.ptglab.com/products/CANX-Antibody-10427-2-AP.htm
33) Validation of Rabbit anti- Phospho-STING (Ser365) (Cell Signaling 62912 clone D1C4T, 1:200): The manufacture states that the specificity of the antibody was tested by Confocal immunofluorescent analysis of Raw 264.7 cells, transfected with poly(dA:dT) or mock transfected. https://www.cellsignal.com/products/primary-antibodies/phospho-sting-ser365-d1c4t-rabbit-mab/62912?site-search-type=Products&N=4294956287&Ntt=phospho-sting+&fromPage=plp
34) Validation of Mouse anti- TGN38 (Santa Cruz sc-166594,1:200): The manufacture states that the specificity of the antibody was tested by Confocal immunofluorescent analysis and western blot on various mammalian cell. https://www.scbt.com/p/tgn38-antibody-b-6?requestFrom=search
35) Validation of Mouse anti- CD63 (Santa Cruz sc-5275,1:200): The manufacture states that the specificity of the antibody was tested by western blot on various mammalian cell. https://www.scbt.com/p/cd63-antibody-mx-49-129-5?requestFrom=search
36) Validation of Mouse anti- LAMP1 (DSHB 1D4B, 1:50): The manufacture states that the specificity of the antibody was tested by western blot on various mammalian cell. https://dshb.biology.uiowa.edu/1D4B
37) Validation of Mouse agarose-conjugated anti-FLAG (Sigma A4596): The manufacture states that the antibody specifically detected free N-terminal of FALG sequence(N-Asp-Tyr-Lys-Asp-Asp-Asp-Asp-Lys-C). https://www.sigmaaldrich.com/US/en/product/

sigma/a4596
38) Validation of Mouse agarose-conjugated anti-Myc (Sigma 16-219 clone 4A6): The manufacture states that the antibody specifically detected Myc antigen (MEQKLISEEDL). https://www.sigmaaldrich.com/US/en/product/mm/16219
38) Validation of PE anti- Mouse CD4 (BioLegend 100408 clone GK1.5): The manufacture states that the antibody was tested by Flow cytometric analysis of C57BL/6 mouse splenocytes. https://www.biolegend.com/en-us/products/fitc-anti-mouse-cd4-antibody-248
39) Validation of anti- Rat CD8 (Thermo Fisher MA5-17605 clone YTS169.4): The manufacture states that the antibody was tested by Flow cytometric analysis of Mouse Thymus cells. https://www.thermofisher.cn/cn/zh/antibody/product/CD8-alpha-Antibody-clone-YTS169-4-Monoclonal/MA5-17605
40) Validation of anti-Rat F4/80 Antibody (BioLegend 123116, 123114 clone BM8): The manufacture states that the antibody was tested by Flow cytometric analysis of thioglycolate-elicited BALB/c mouse peritoneal macrophages. https://www.biolegend.com/en-us/products/pe-cyanine7-anti-mouse-f4-80-antibody-4070
41) Validation of anti-Rat CD11b (BioLegend 101206 clone M1/70): The manufacture states that the antibody was tested by Flow cytometric analysis of C57BL/6 mouse bone marrow cells. https://www.biolegend.com/en-us/products/fitc-anti-mouse-human-cd11b-antibody-347
42) Validation of anti-Rat Gr-1 (BioLegend 108408 clone RB6-8C5): The manufacture states that the antibody was tested by Flow cytometric analysis of C57BL/6 mouse bone marrow cells. https://www.biolegend.com/en-us/products/pe-anti-mouse-ly-6g-ly-6c-gr-1-antibody-460
43) Validation of anti-Armenian Hamster TCR β (BioLegend 109206 clone H57-597): The manufacture states that the antibody was tested by Flow cytometric analysis of C57BL/6 mouse splenocytes. https://www.biolegend.com/en-us/products/fitc-anti-mouse-tcr-beta-chain-antibody-270
44) Validation of anti-Rat B220 (BioLegend 103206, 103208 clone RA3-6B2): The manufacture states that the antibody was tested by Flow cytometric analysis of C57BL/6 mouse splenocytes. https://www.biolegend.com/en-us/products/fitc-anti-mouse-human-cd45r-b220-antibody-445, https://www.biolegend.com/en-us/products/pe-anti-mouse-human-cd45r-b220-antibody-447
45) Validation of anti-Rat CD45 (BioLegend 103130 clone 30-F11): The manufacture states that the antibody was tested by Flow cytometric analysis of C57BL/6 mouse splenocytes. https://www.biolegend.com/en-us/products/percp-anti-mouse-cd45-antibody-4265
46) Validation of anti-Rat I-A/I-E (BioLegend 107645, 107608 clone M5/114.15.2): The manufacture states that the antibody was tested by Flow cytometric analysis of C57BL/6 mouse splenocytes. https://www.biolegend.com/en-us/products/brilliant-violet-785-anti-mouse-i-a-i-e-antibody-12087, https://www.biolegend.com/en-us/products/pe-anti-mouse-i-a-i-e-antibody-367
47) Validation of anti-Mouse H-2Kb/H-2Db (BioLegend 114606 clone 28-8-6; 116506 clone AF6-88.5): The manufacture states that the antibody was tested by Flow cytometric analysis of
C57BL/6 mouse splenocytes. https://www.biolegend.com/en-us/products/fitc-anti-mouse-h-2kb-h-2db-antibody-1683; https://www.biolegend.com/en-us/products/fitc-anti-mouse-h-2kb-antibody-1748
48) Validation of anti-Rat TLR2 (BioLegend 148604 clone CB225): The manufacture states that the antibody was tested by Flow cytometric analysis of thioglycolate-elicited BALB/c mouse peritoneal macrophages. https://www.biolegend.com/en-us/products/pe-anti-mouse-cd282-tlr2-antibody-10230
49) Validation of anti-Rat TLR4 (BioLegend 145406 clone SA15-21): The manufacture states that the antibody was tested by Flow cytometric analysis of thioglycolate-elicited BALB/c mouse peritoneal macrophages. https://www.biolegend.com/en-us/products/apc-anti-mouse-cd284-tlr4-antibody-8871
50) Validation of anti-Rat PD-L1 (BioLegend 124308 clone 10F.9G2): The manufacture states that the antibody was tested by Flow cytometric analysis of C57BL/6 mouse splenocytes. https://www.biolegend.com/en-us/products/pe-anti-mouse-cd274-b7-h1-pd-l1-antibody-4497
51) Validation of anti-Rat CD16/CD32 (BioLegend 101302 clone 93): The manufacture states that the antibody was tested by Flow cytometric analysis of C57BL/6 mouse splenocytes. https://www.biolegend.com/en-us/products/purified-anti-mouse-cd16-32-antibody-190

Eukaryotic cell lines

Policy information about cell lines and Sex and Gender in Research

Cell line source(s)

MEF and RAW 264.7 cell lines were originally obtained from ATCC; DN32.D3 cell line was a gift from Dr. Mitchell Kronenberg (La Jolla Institute for Immunology); PDAC cell line was a gift from Dr. Raghu Kalluri (MD Anderson Cancer Center, Houston, TX, USA). Vero Cells were provided by Dr. Malini Raghavan at University of Michigan Medical School.

Authentication

The cells have been authenticated by morphology. Expression and secretion of inflammatory cytokines upon ligand stimulation was confirmed for MEF, RAW 264.7 and DN32.D3. PDAC cells form tumors in mice with expected tumor morphology and characteristics. High HSV-1 susceptibility of Vero Cells were confirmed, consistent with absence of type I IFN in this cell line. Authentication was done before the experiments during this study.

Mycoplasma contamination

All cell lines used for experiments had no mycoplasma contamination after testing.

Commonly misidentified lines
(See ICLAC register)

These cell lines are not listed in that database.

Animals and other research organisms

Policy information about studies involving animals; ARRIVE guidelines recommended for reporting animal research, and Sex and Gender in Research

Laboratory animals

All genetically engineered mice were in C57BL/6J background. Nude mice are on the BALB/c background. 8-week-old male Sel1LLyz2 and Sel1Lf/f mice were used for high-fat diet feeding for up to 20 weeks. For in vivo tumor study, 6-8 week-old male mice were used for tumor cell implantation. For LPS Challenge in vivo, 8 w-old female Sel1LLyz2 and Sel1Lf/f mice were used. For collection of primary macrophage and T cells for in vitro study, 2-4 months-old, male and female Sel1LLyz2, Sel1Lf/f, Atg7Lyz2, Atg7f/f and OT1

mice were used. The background, gender and age of mice are specifically indicated in the text and legends.

Wild animals	N/A
Reporting on sex	Both males and females were used in this study, and results are applicable for both genders.
Field-collected samples	No field-collected samples used in this study.
Ethics oversight	All animal procedures were approved by and done in accordance with the Institutional Animal Care and Use Committee (IACUC) at the University of Michigan Medical School (PRO00008989) and Cornell University (#2007-0051), and Animal Experimentation Ethics Committee of the First Affiliated Hospital, Zhejiang University School of Medicine (#2021-0135).

Note that full information on the approval of the study protocol must also be provided in the manuscript.

Flow Cytometry

Plots

Confirm that:

☒ The axis labels state the marker and fluorochrome used (e.g. CD4-FITC).

☒ The axis scales are clearly visible. Include numbers along axes only for bottom left plot of group (a 'group' is an analysis of identical markers).

☒ All plots are contour plots with outliers or pseudocolor plots.

☒ A numerical value for number of cells or percentage (with statistics) is provided.

Methodology

Sample preparation	Following incubation with anti-CD16/CD32 antibody to block Fc receptors, 1 million cells were incubated with 20 µl of antibodies diluted at optimal concentrations (at 1:100 or 200) for 20 min at 4°C. Cells were washed three times with PBS and then resuspended in 200 µl PBS for analysis. For intracellular staining, cells were fixed after surface staining and permeabilized with a BD Cytofix/Cytoperm fixation/permeabilization kit according to the manufacturer's protocol. Purification of stromal vascular cells (SVC) from epididymal fat pads followed by flow cytometric analysis were performed as previously described (80).<br>The information is detailed in the methods under the section of 'Flow cytomytric analysis of cell surface markers''.
Instrument	Samples were collected using BD LSR cell analyzer.
Software	Sample are collected by using the FACSDiva v6.2 software (BD Biosciences) and data are analyzed with using FACSDiva v6.2 and Flowjo 8.7 (Flowjo.com).
Cell population abundance	At least 10,000 live splenocytes and 100,000 stromal vascular cells were collected for FACS analysis. Relevant cells sorted from mouse were re-run on Flow cytometry to ensure purity.
Gating strategy	Gates and boundaries were clearly visible in the figures.

☒ Tick this box to confirm that a figure exemplifying the gating strategy is provided in the Supplementary Information.
