# Supplementary figures and images for: SEL1L–HRD1 endoplasmic reticulum-associated degradation controls STING-mediated innate immunity by limiting the size of the activable STING pool
Source: Nat Cell Biol. 2023 May 4;25(5):726–39. doi: 10.1038/s41556-023-01138-4 (PMC10185471; doi:10.1038/s41556-023-01138-4)

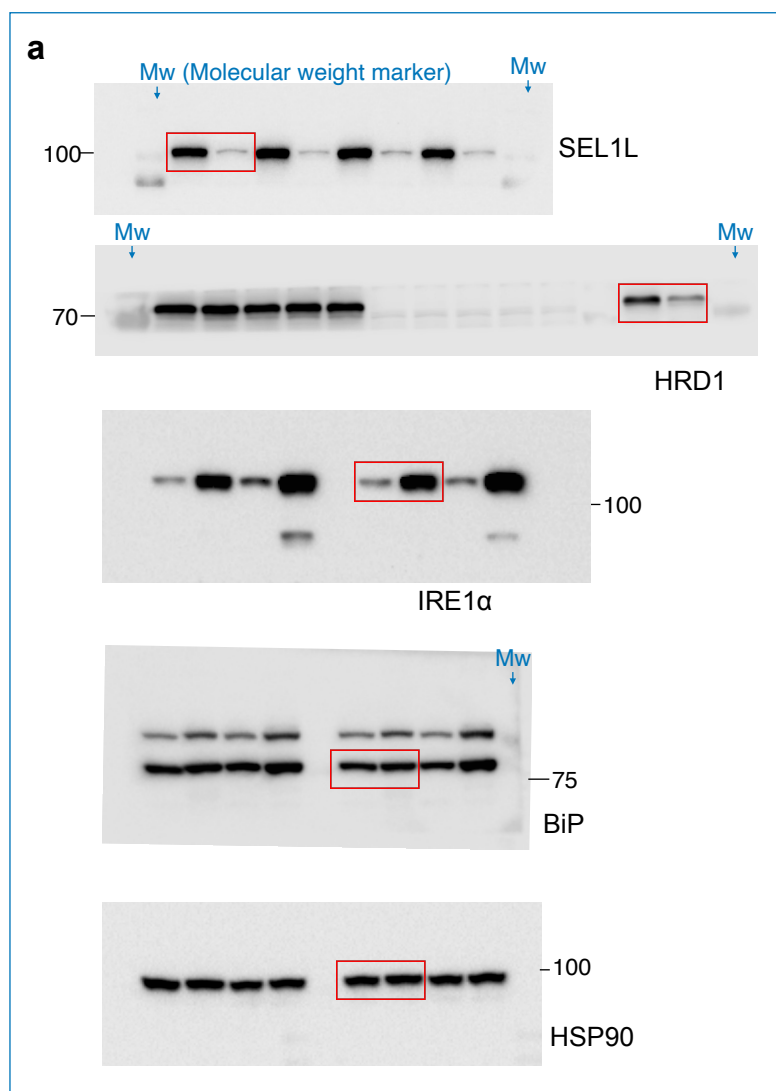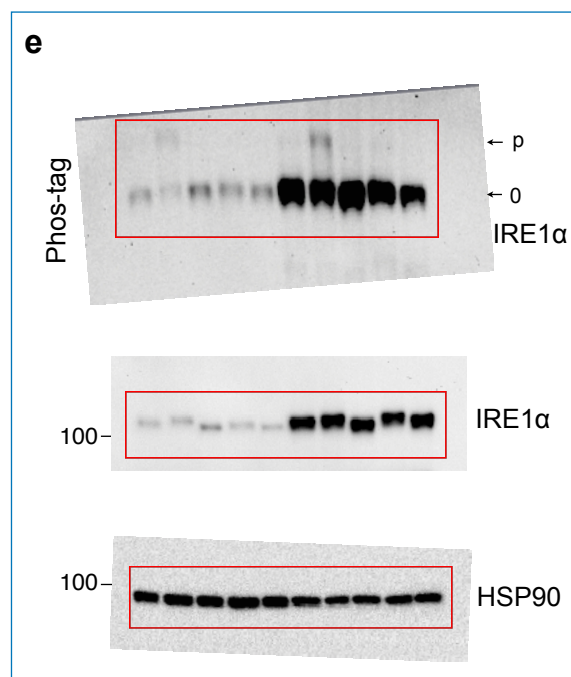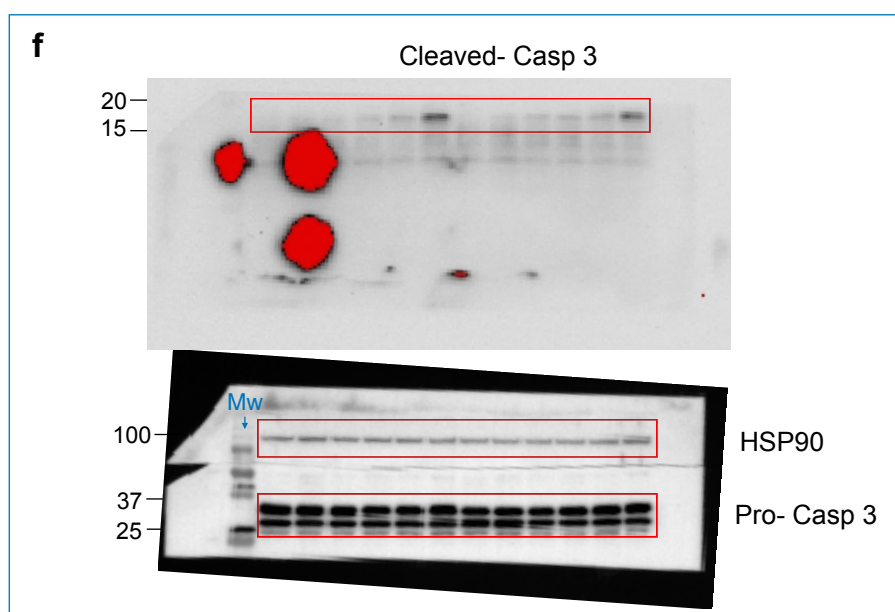

Uncropped immunoblot images of Fig. 1

Supplement: Source Data Fig. 1 — Unprocessed western blots. [file 41556_2023_1138_MOESM3_ESM.pdf]

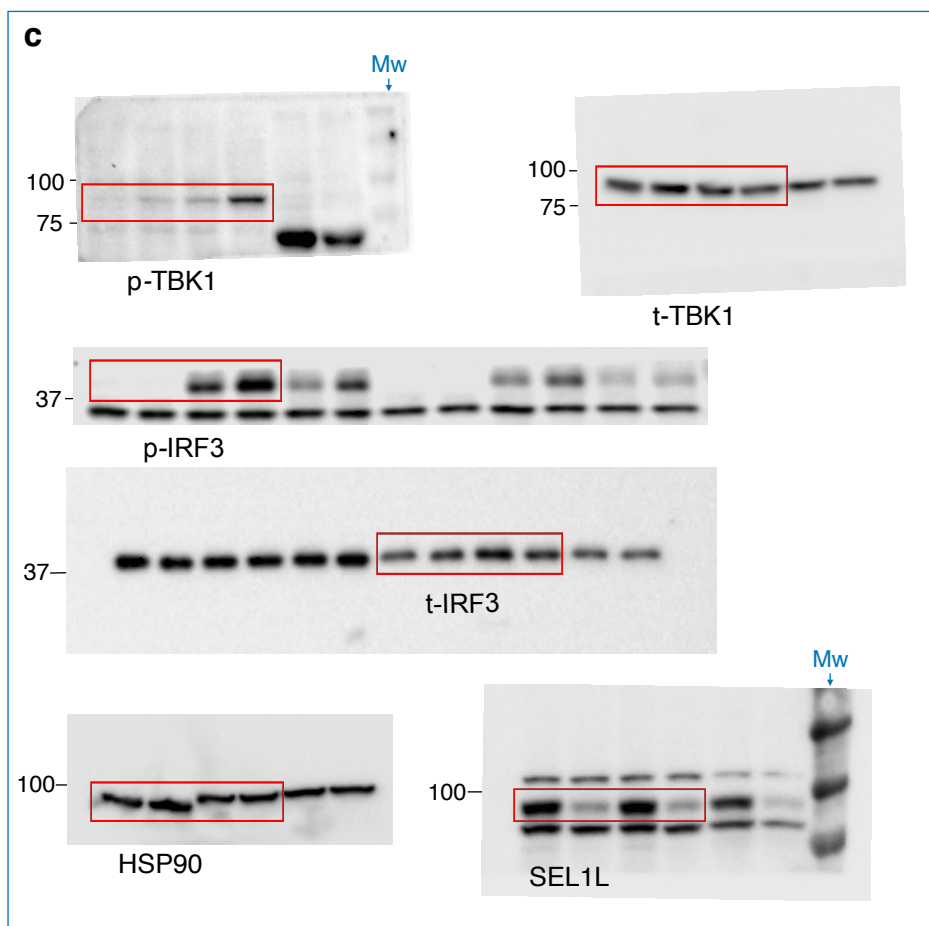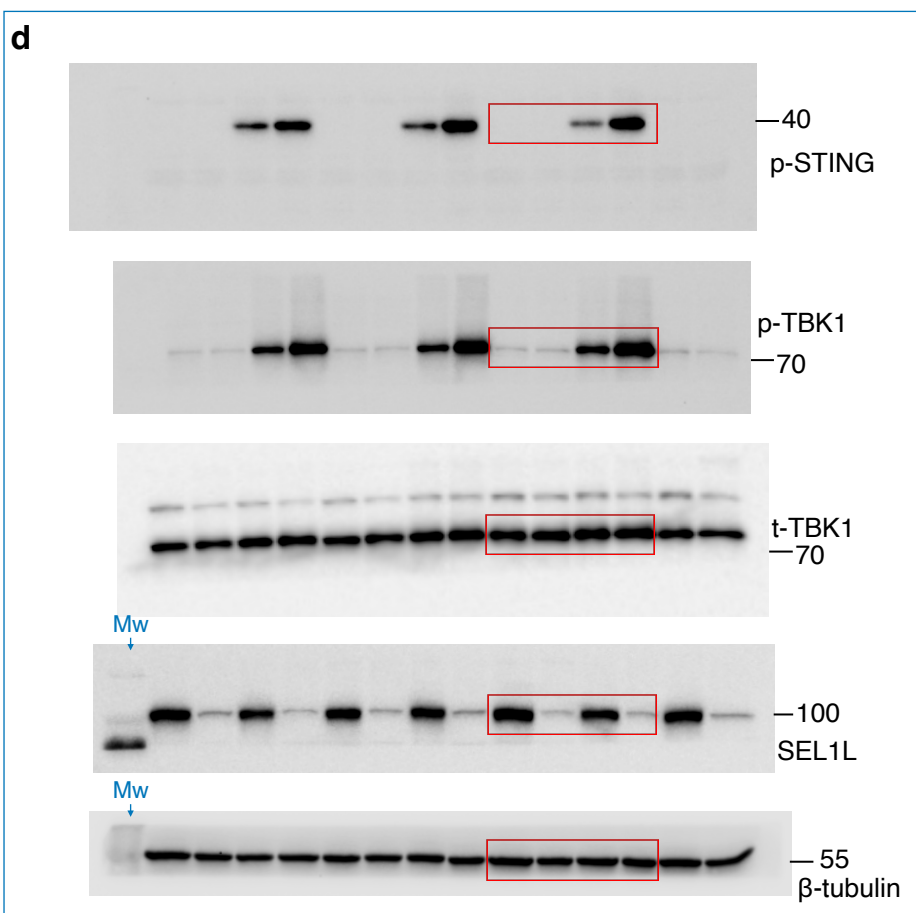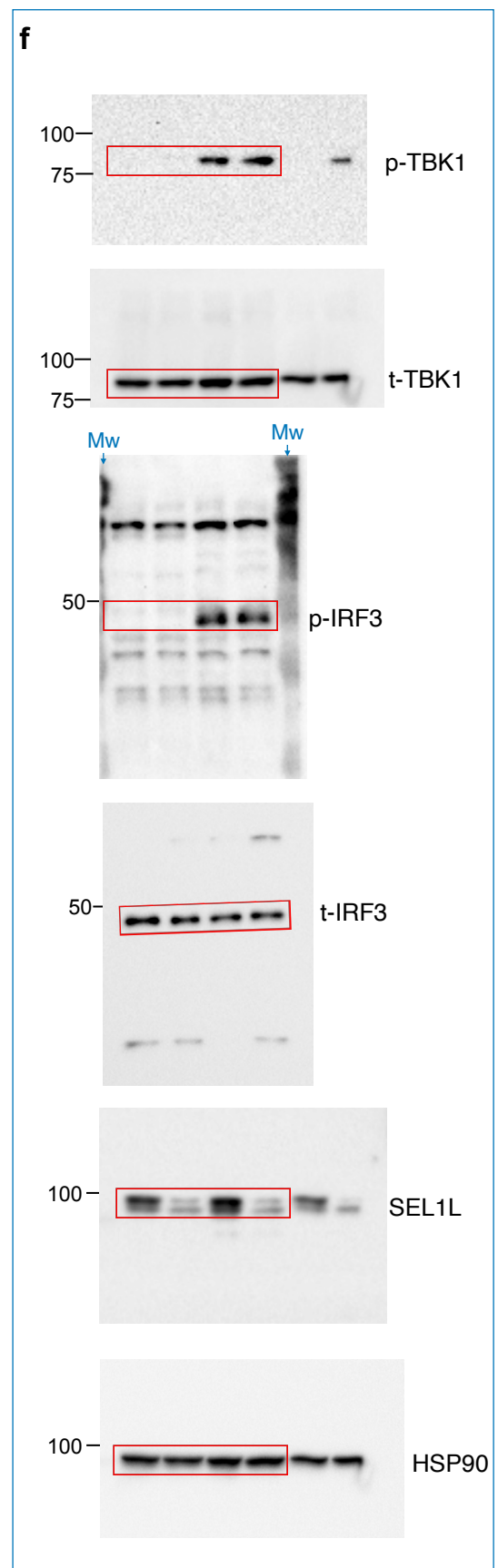

Uncropped immunoblot images of Fig. 2

Supplement: Source Data Fig. 2 — Unprocessed western blots. [file 41556_2023_1138_MOESM5_ESM.pdf]

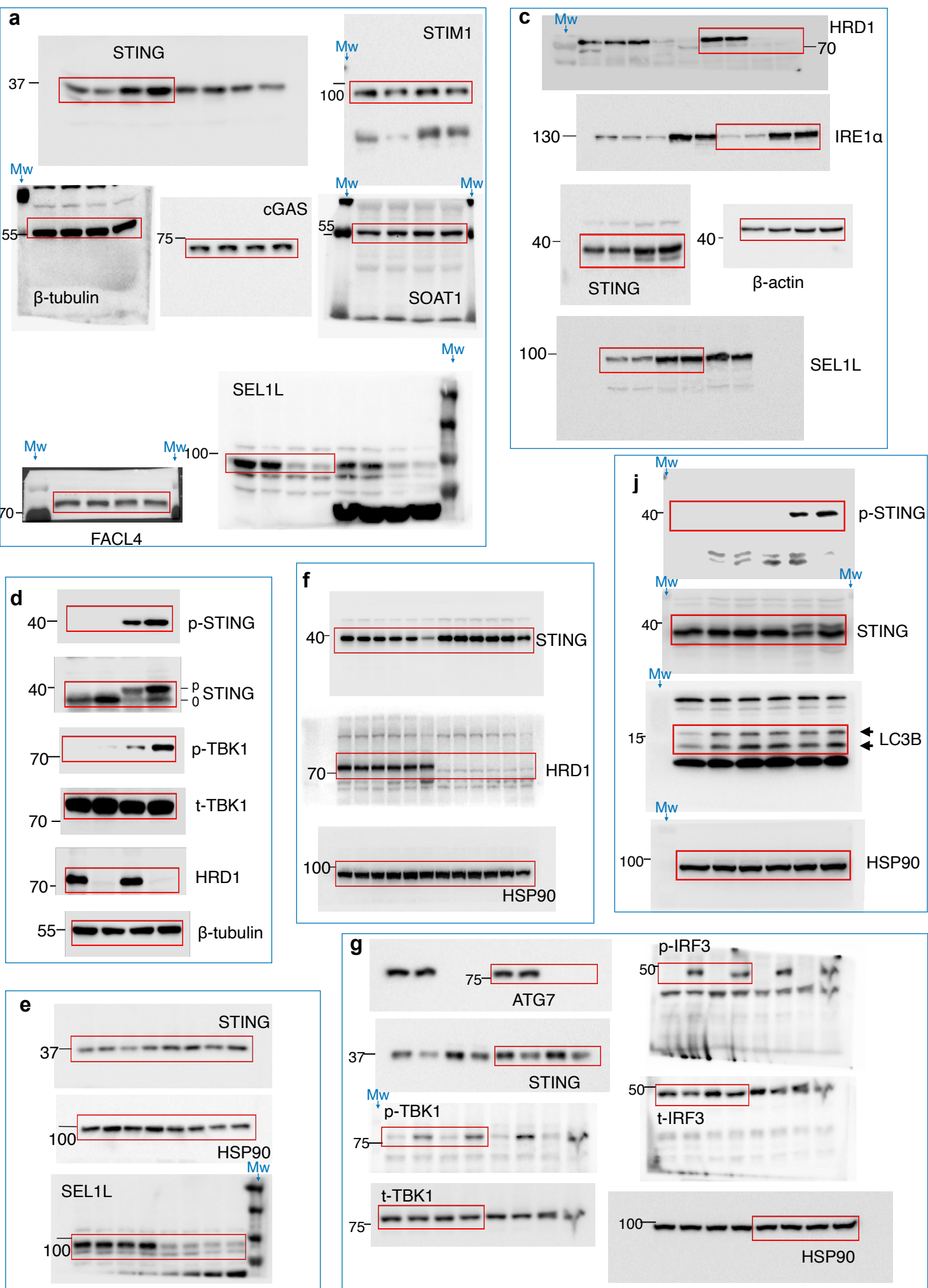

Uncropped immunoblot images of Fig. 3

Supplement: Source Data Fig. 3 — Unprocessed western blots. [file 41556_2023_1138_MOESM7_ESM.pdf]

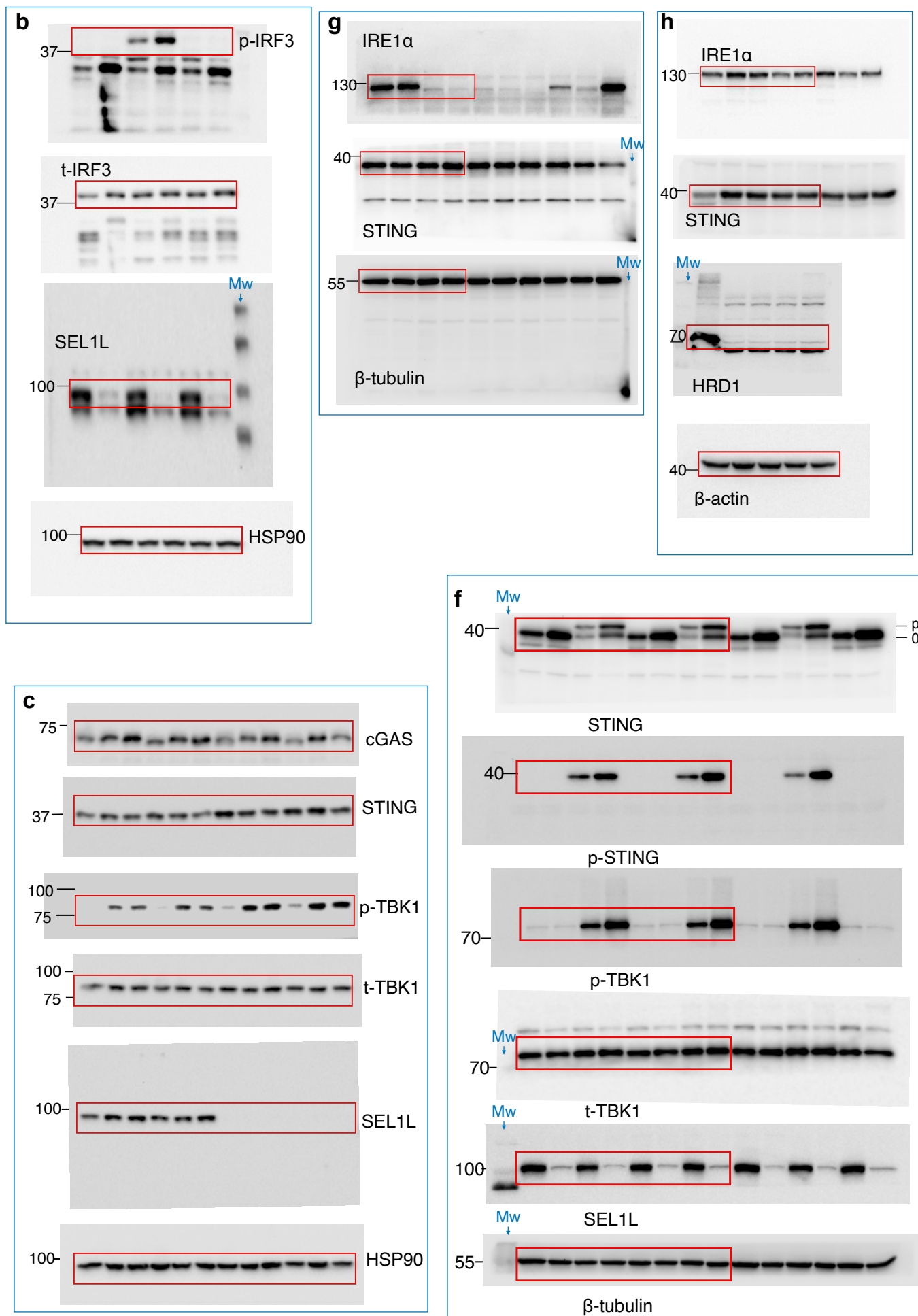

Uncropped immunoblot images of Fig. 4

Supplement: Source Data Fig. 4 — Unprocessed western blots. [file 41556_2023_1138_MOESM9_ESM.pdf]

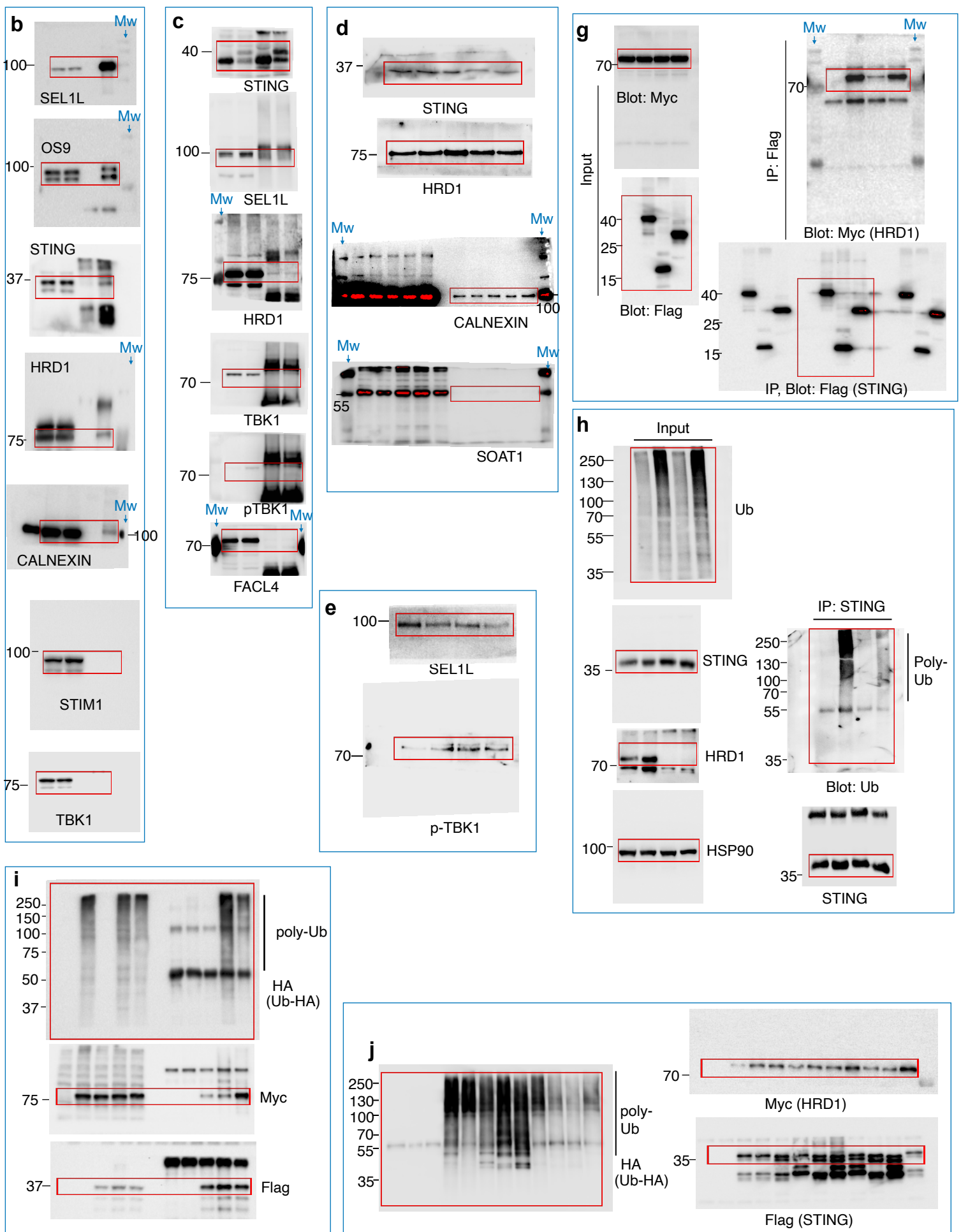

Uncropped immunoblot images of Fig. 5

Supplement: Source Data Fig. 5 — Unprocessed western blots. [file 41556_2023_1138_MOESM11_ESM.pdf]

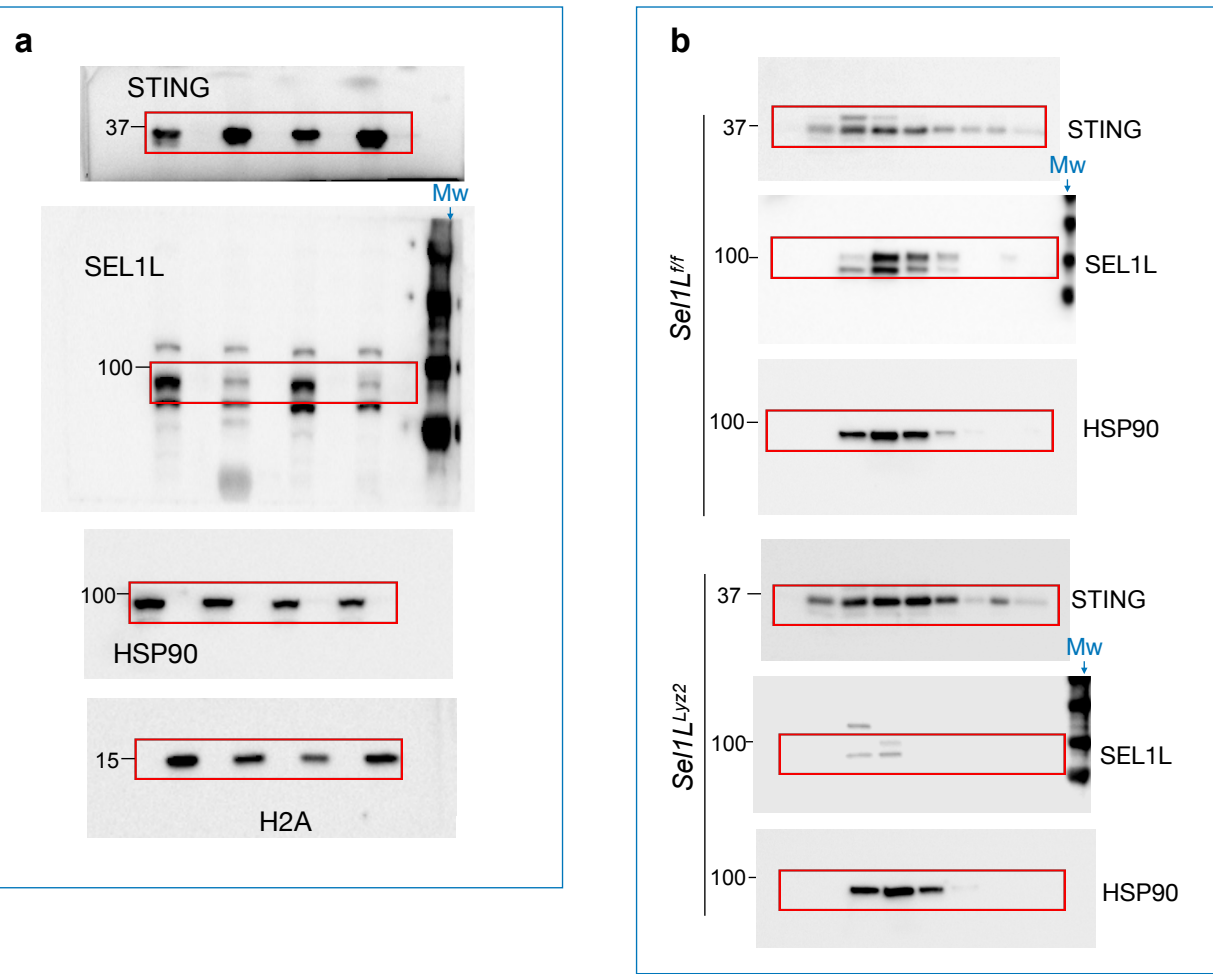

Uncropped immunoblot images of Fig. 6

Supplement: Source Data Fig. 6 — Unprocessed western blots. [file 41556_2023_1138_MOESM13_ESM.pdf]

**a**

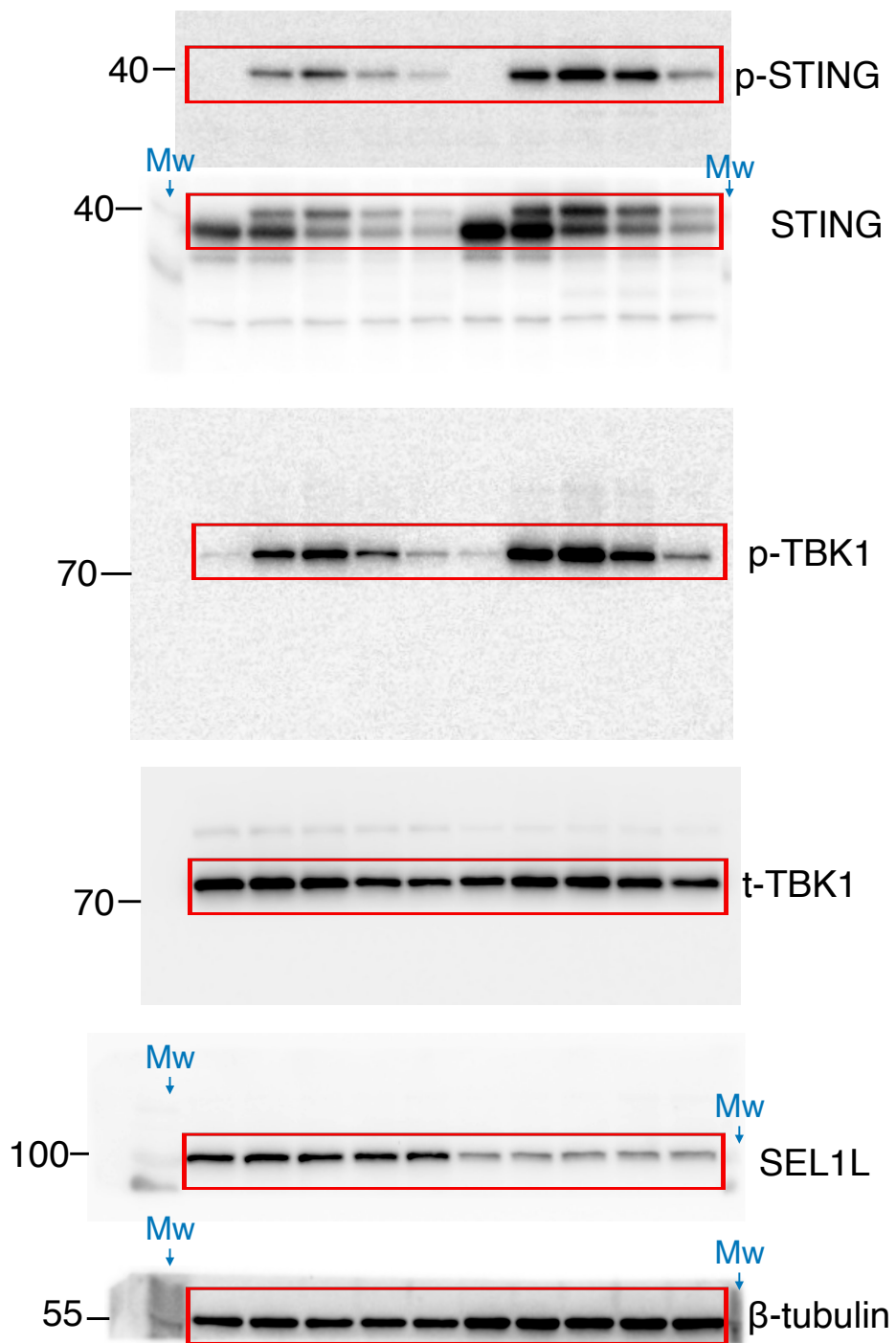

**Uncropped immunoblot images of Fig. 7**

Supplement: Source Data Fig. 7 — Unprocessed western blots. [file 41556_2023_1138_MOESM15_ESM.pdf]

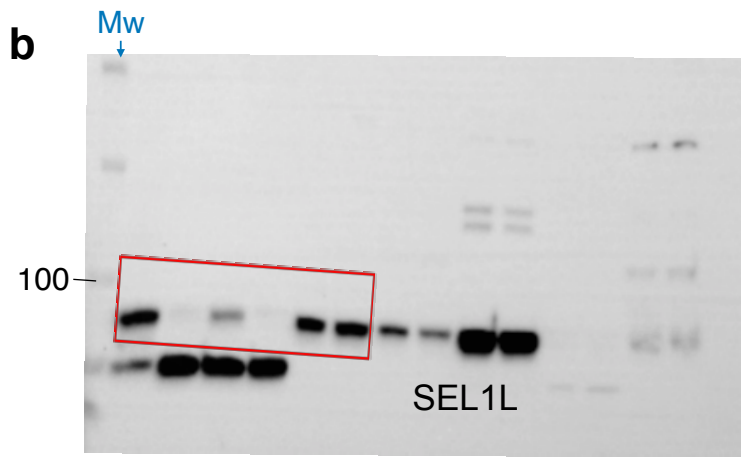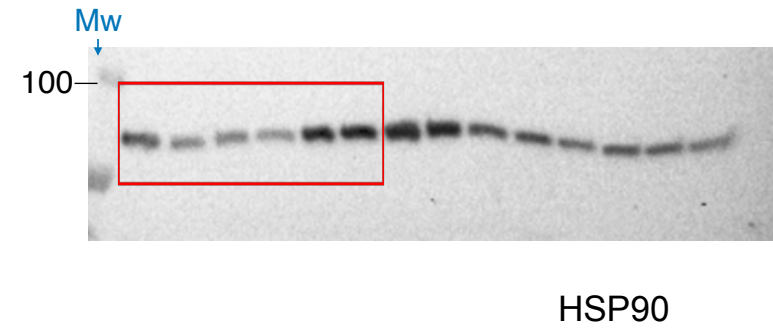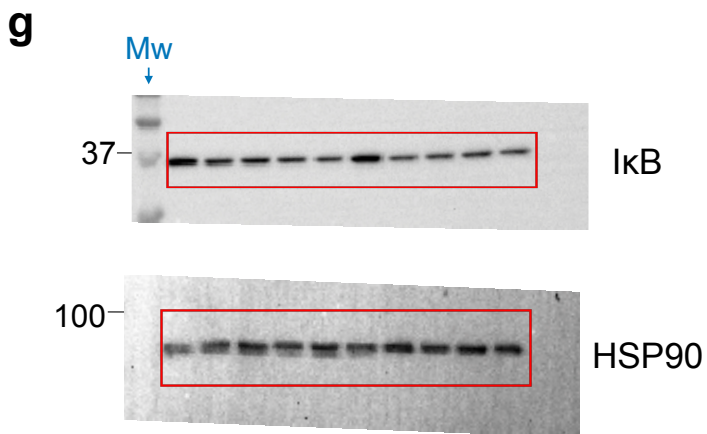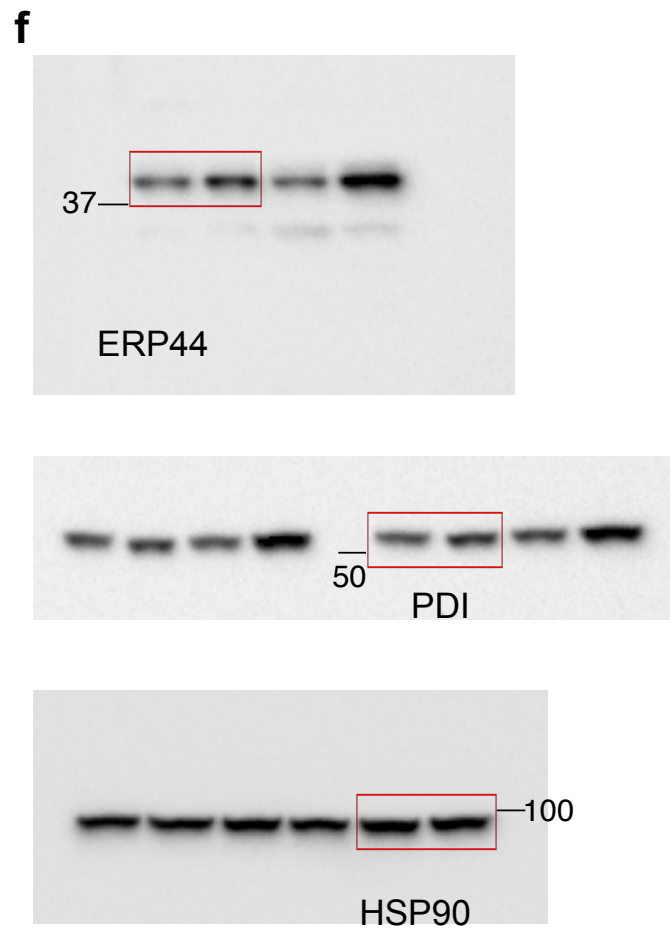

**Uncropped immunoblot images of Extended Data Fig. 1**

Supplement: Source Data Extended Data Fig. 1 — Unprocessed western blots. [file 41556_2023_1138_MOESM17_ESM.pdf]

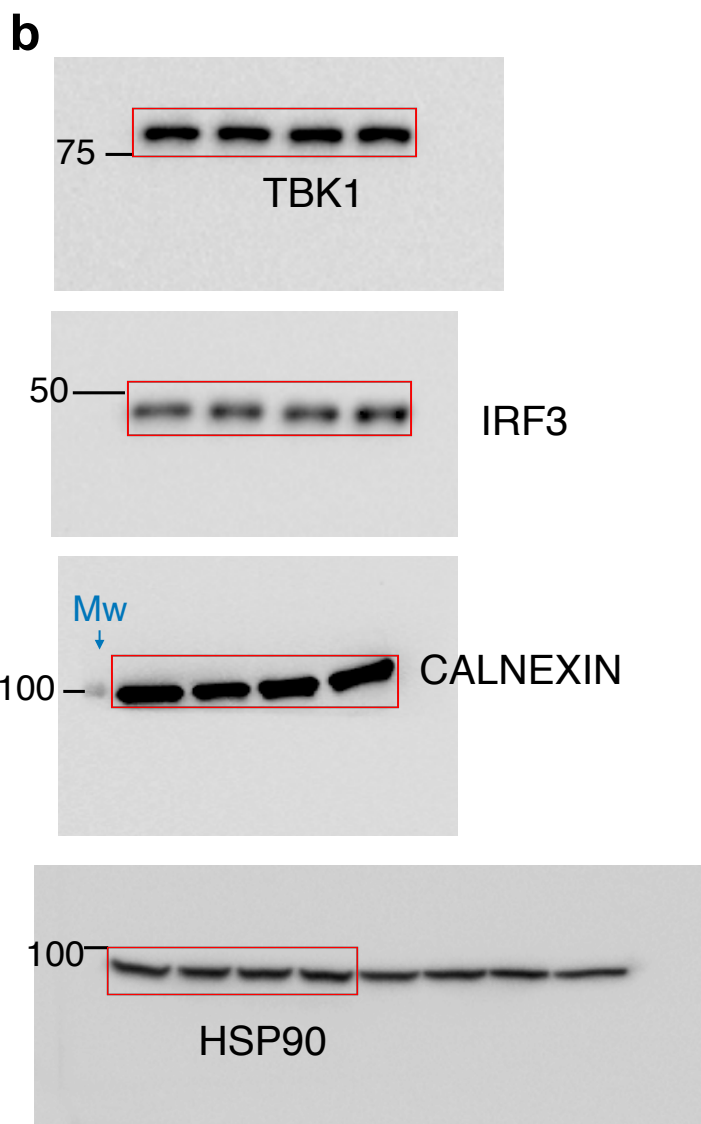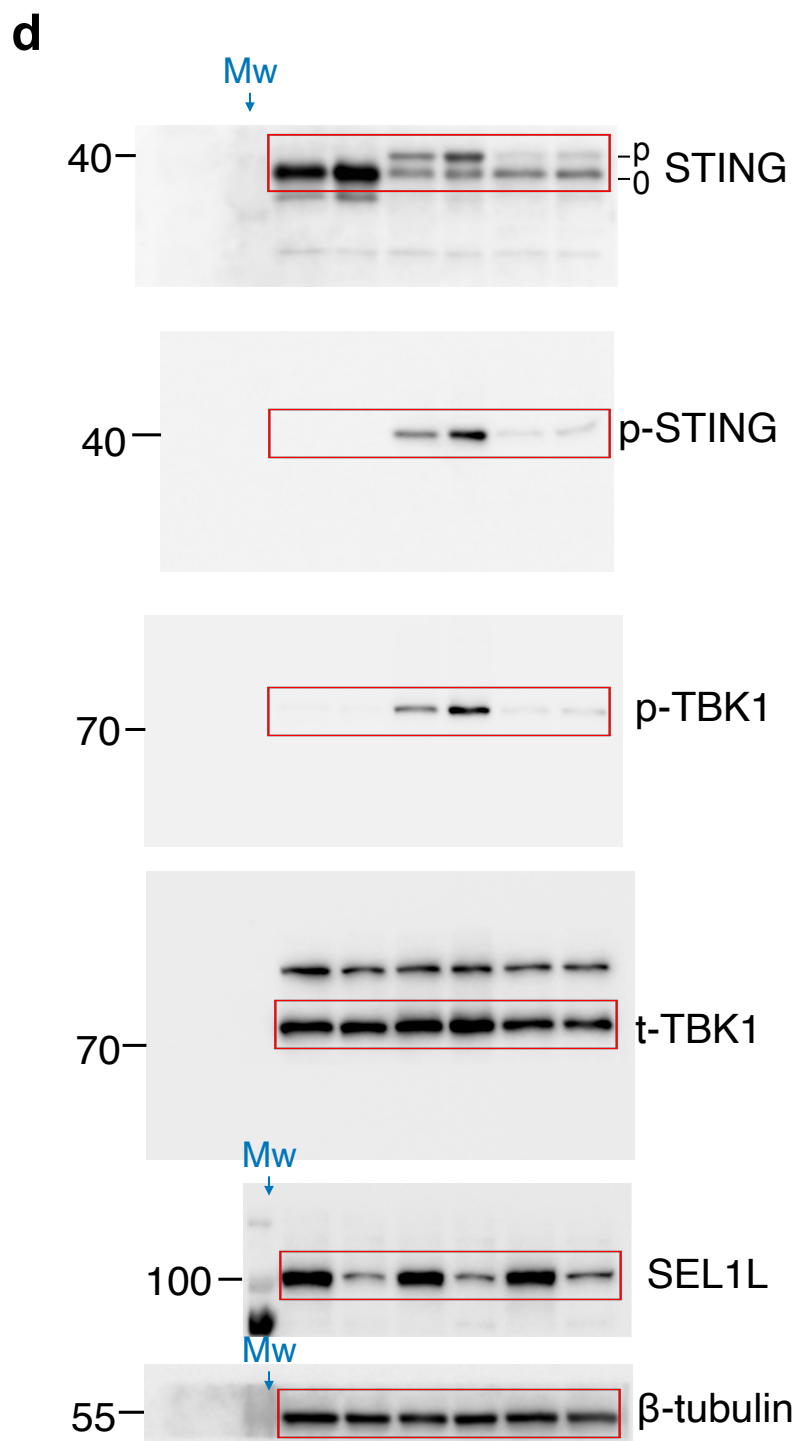

**Uncropped immunoblot images of Extended Data Fig. 3**

Supplement: Source Data Extended Data Fig. 3 — Unprocessed western blots. [file 41556_2023_1138_MOESM20_ESM.pdf]

**a**

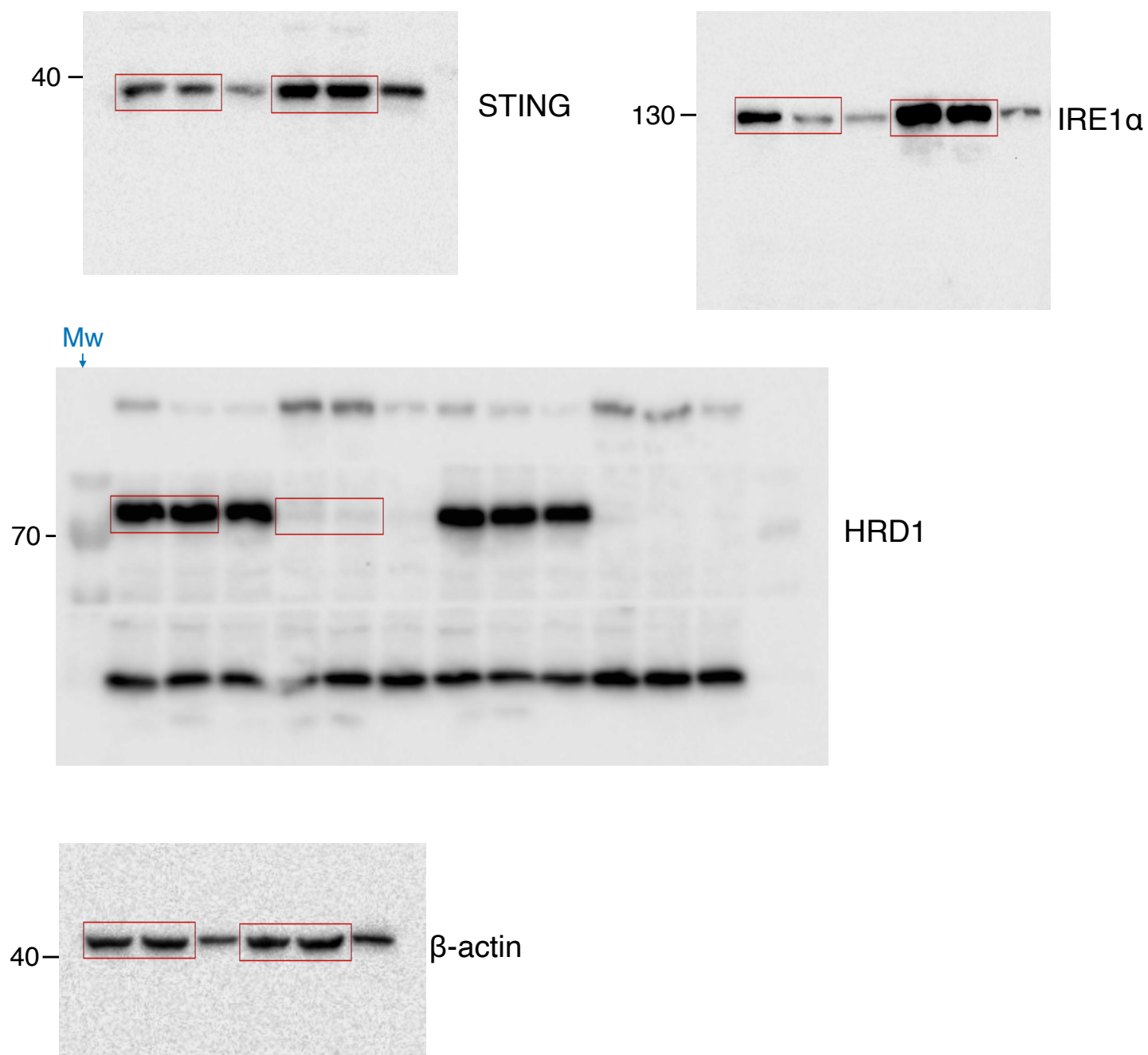

**Uncropped immunoblot images of Extended Data Fig. 4**

Supplement: Source Data Extended Data Fig. 4 — Unprocessed western blots. [file 41556_2023_1138_MOESM22_ESM.pdf]

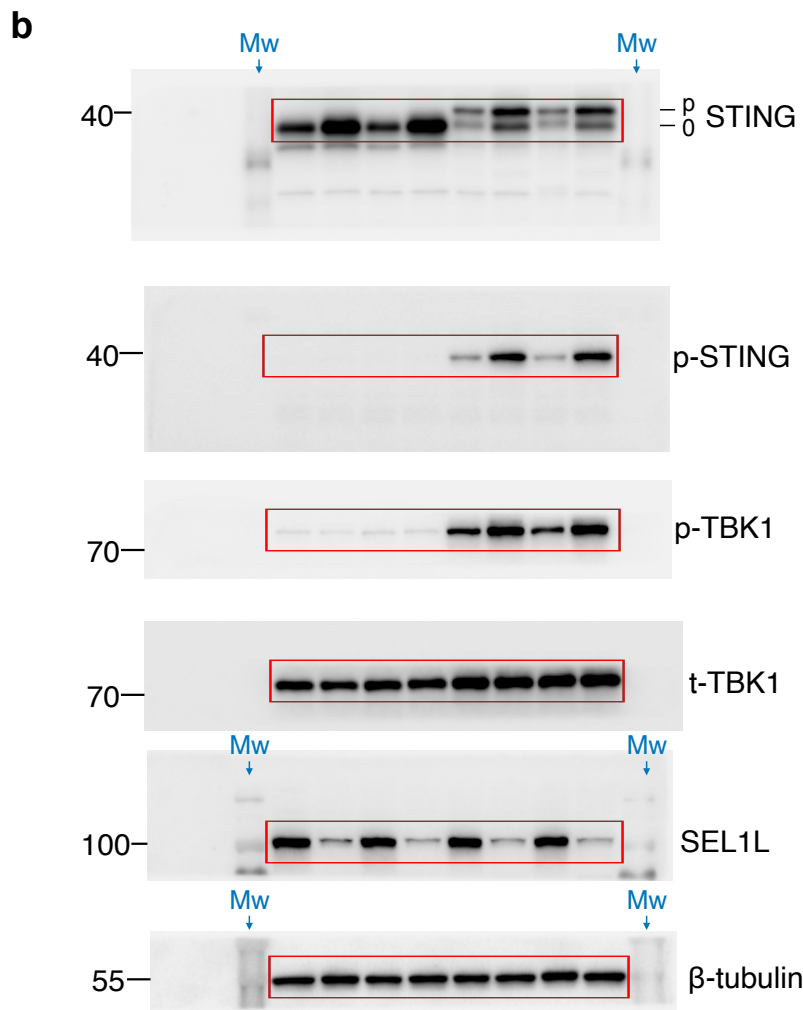

**Uncropped immunoblot images of Extended Data Fig. 5**

Supplement: Source Data Extended Data Fig. 5 — Unprocessed western blots. [file 41556_2023_1138_MOESM24_ESM.pdf]

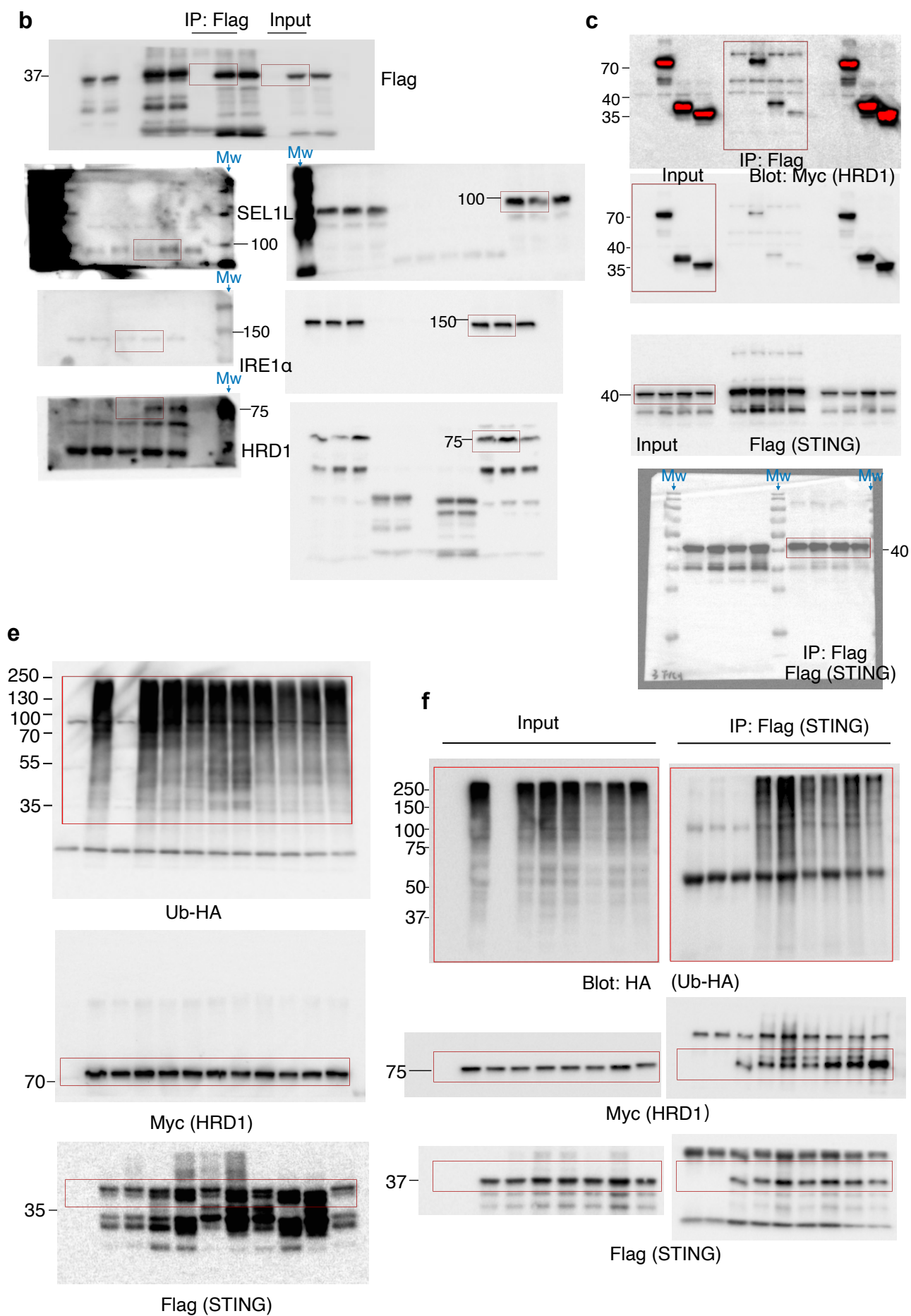

**Uncropped immunoblot images of Extended Data Fig. 6**

Supplement: Source Data Extended Data Fig. 6 — Unprocessed western blots. [file 41556_2023_1138_MOESM26_ESM.pdf]

**a**

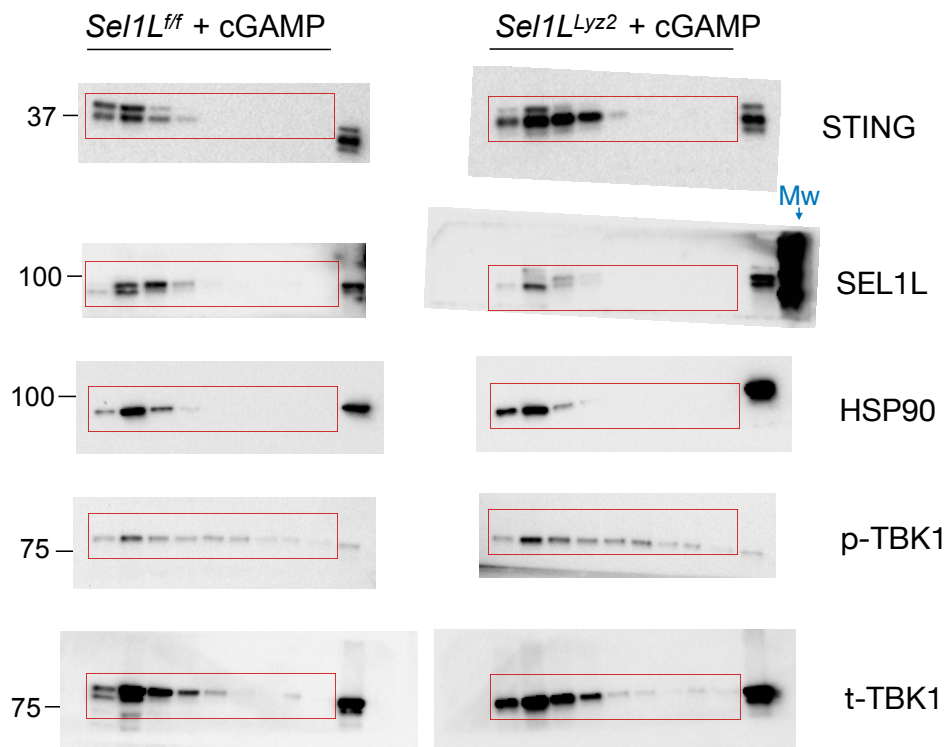

**Uncropped immunoblot images of Extended Data Fig. 7**

Supplement: Source Data Extended Data Fig. 7 — Unprocessed western blots. [file 41556_2023_1138_MOESM28_ESM.pdf]

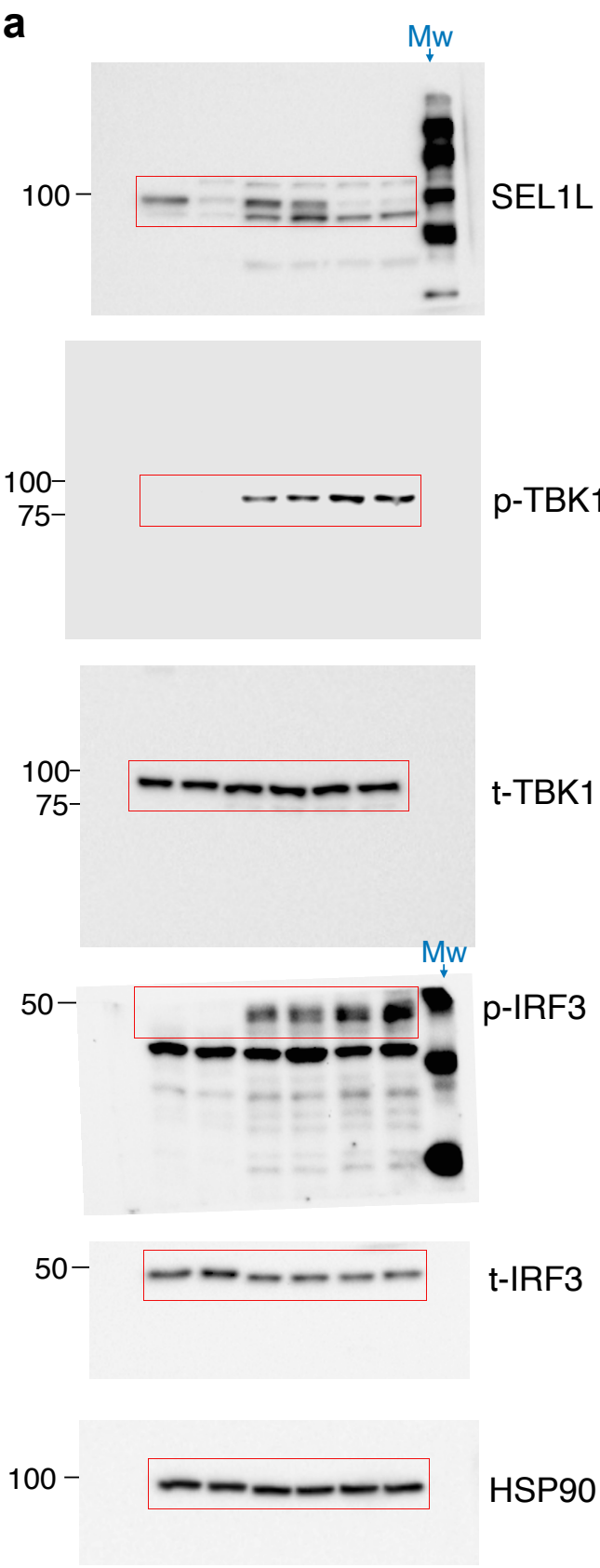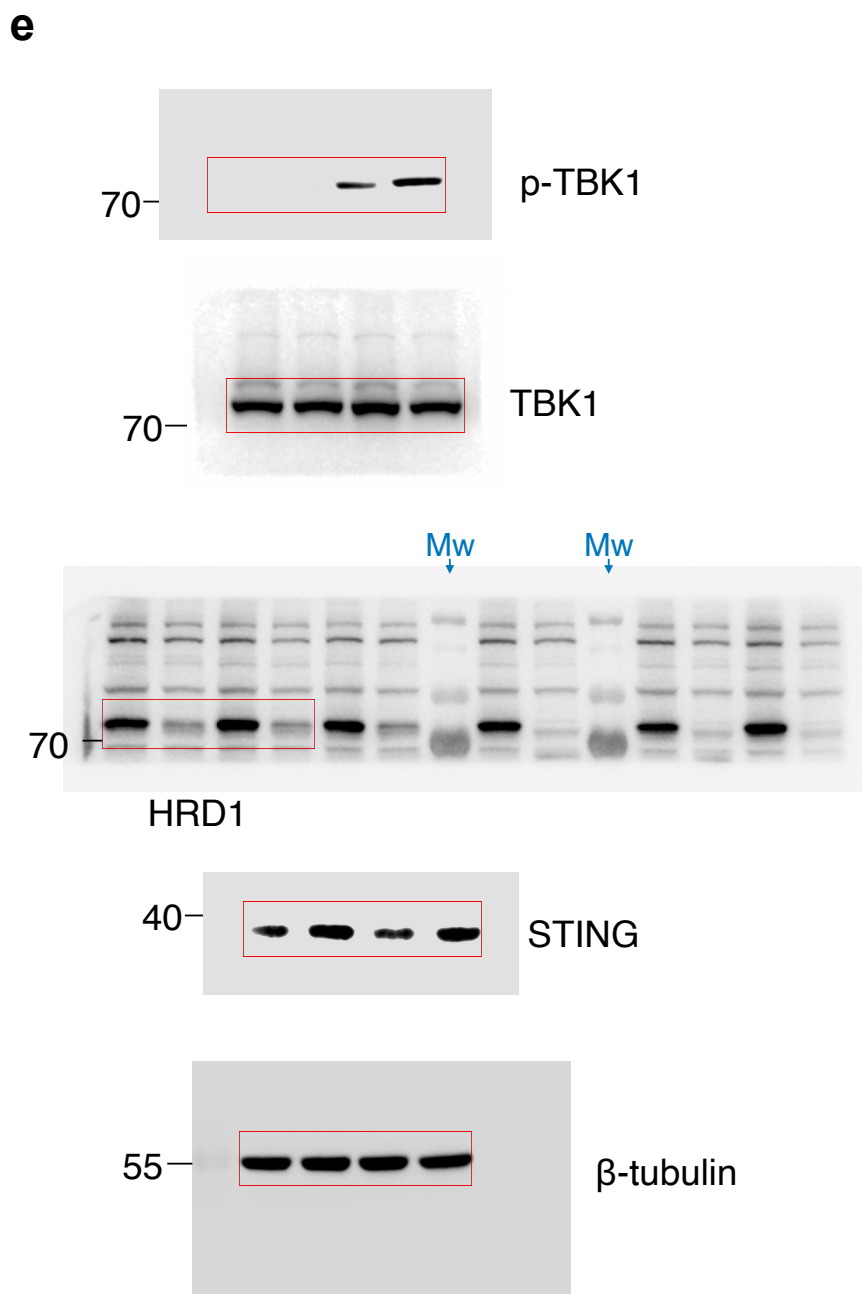

Uncropped immunoblot images of Extended Data Fig. 8

Supplement: Source Data Extended Data Fig. 8 — Unprocessed western blots. [file 41556_2023_1138_MOESM30_ESM.pdf]
